# S100A13 promotes senescence-associated secretory phenotype and cellular senescence via modulation of non-classical secretion of IL-1α

**DOI:** 10.18632/aging.101760

**Published:** 2019-01-23

**Authors:** Yuanyuan Su, Chenzhong Xu, Zhaomeng Sun, Yao Liang, Guodong Li, Tanjun Tong, Jun Chen

**Affiliations:** ^1^ Peking University Research Center on Aging, Beijing Key Laboratory of Protein Posttranslational Modifications and Cell Function, Department of Biochemistry and Molecular Biology, Peking University Health Science Center, Beijing 100191, China

**Keywords:** S100A13, non-classical protein secretory pathway, IL-1α, SASP, Cu^2+^, cell senescence

## Abstract

Senescent cells display the senescence-associated secretory phenotype (SASP) which plays important roles in cancer, aging, etc. Cell surface-bound IL-1α is a crucial SASP factor and acts as an upstream regulator to induce NF-κB activity and subsequent SASP genes transcription. IL-1α exports to cell surface via S100A13 protein-dependent non-classical secretory pathway. However, the status of this secretory pathway during cellular senescence and its role in cellular senescence remain unknown. Here, we show that S100A13 is up-regulated in various types of cellular senescence. S100A13 overexpression increases cell surface-associated IL-1α level, NF-κB activity and subsequent multiple SASP genes induction, whereas S100A13 knockdown has an opposite role. We also exhibit that Cu^2+^ level is elevated during cellular senescence. Lowering Cu^2+^ level decreases cell surface-bound IL-1α level, NF-κB activity and SASP production. Moreover, S100A13 overexpression promotes oncogene Ras-induced cell senescence (Ras OIS), Doxorubicin-induced cancer cell senescence (TIS) and replicative senescence, while impairment of non-classical secretory pathway of IL-1α delays cellular senescence. In addition, intervention of S100A13 affects multiple SASP and cellular senescence mediators including p38, γ-H2AX, and mTORC1. Taken together, our findings unveil a critical role of the non-classical secretory pathway of IL-1α in cellular senescence and SASP regulation.

## INTRODUCTION

Cellular senescence is a permanent cell cycle arrest state in response to various intracellular and extracellular stimuli such as telomere erosion because of repeated cell division (replicative senescence), DNA damage, oxidative stress, and oncogenes including Ras or Myc activation, etc [1]. One hallmark of senescence is that senescent cells secret multiple pro-inflammatory cytokines, chemokines, growth factors, and other proteins which is referred to as senescence-associated secretory phenotype (SASP) [1]. The SASP has been shown to have context-dependent pleiotropic biological and physiological functions. For instance, SASP has tumor suppressive roles either via cell autonomous mechanism to reinforce cell senescence [2], or using ‘immune surveillance’ mechanism via cell non-autonomous fashion [3]. The SASP factors also assist tissue repair, embryonic development, as well as in vivo cell reprogramming through paracrine manner [4–6]. However, the mounting evidences also show that SASP factors can promote tumor growth and invasion, and contribute to many age-related diseases and aging in late-life [7].

Two transcription factors NF-κB and C/EBPβ are required for the SASP genes transcription [2, 8]. The persistent activation of ATM/ATR-CHK1/CHK2-mediated DNA damage response (DDR) pathway [9], and p38α MAPK-mediated stress response pathway [10] are reported to regulate NF-κB activity and SASP genes expression independently. Cell surface-bound IL-1α is an upstream regulator of SASP genes expression by feed forward inducing NF-κB activity [11]. The DDR-dependent activation of transcription factor GATA4 has also been reported to regulate NF-κB activity and SASP genes induction [12]. More recently, it has been shown that the innate immunity cytosolic DNA-sensing cGAS–STING pathway is essential for SASP genes induction by stimulating NF-κB activity [13–15].

SASP factors exert their functions via either autocrine or paracrine manner. In general, most SASP factors are secreted to extracellular compartment via classical endoplasmic reticulum (ER)-Golgi protein secretory pathway [16]. However, a minority of proteins without a hydrophobic signal peptide located usually at the N-terminus, secret to cell surface independent of conventional secretory pathway, which is termed as non-classical secretory pathway [17]. IL-1α, as a crucial SASP factor, secrets to cell membrane surface via the non-classical secretory pathway [17]. First, S100A13, a member of a large gene family of small acidic proteins [18], binds to IL-1α, and constitutes the core component of the multiprotein complex. The combination of these two proteins is the key step in the non-classical secretion of IL-1α [19]. Then, this complex interacts with Cu^2+^ ions and migrates close to the acidic environment of the inner leaflet of the cell membrane [20, 21]. Last, IL-1α is secreted to cell surface [21]. During cellular senescence, cell surface-bound IL-1α binds to its receptor IL-1R in a juxtacrine fashion to stimulate NF-κB activity, thus, IL-1α and NF-κB comprise a positive feedback loop and IL-1α acts as an upstream regulator of SASP induction [11]. However, the state of the non-classical secretory pathway of IL-1α during cellular senescence remains unknown, and whether this pathway involves in the SASP induction and cellular senescence has not been defined.

In this study, we present that S100A13 and Cu^2+^, two critical components in mediating the non-classical secretion of IL-1α, play crucial roles in modulating NF-κB activity and SASP expression, as well as cellular senescent response.

## RESULTS

### S100A13 is induced and regulates cell surface-bound IL-1α level during cell senescence

To investigate whether S100A13-dependent non-classical secretory pathway of IL-1α participates in regulating SASP expression, we used IMR90 cells expressing ER:Ras fusion protein (ER:Ras-IMR90 cells) as a oncogene Ras-induced cell senescence model (Ras OIS) which developed strong SASP. It is reported that human colon cancer cells HCT116 can be induced to senescence by low concentration of Doxorubicin treatment, and possess typical senescent cell features such as the persistent DDR, the up-regulation of NF-κB activity and SASP expression which is similar to replicative senescence and other stimuli-induced premature senescence [22]. Therefore, we took this advantage to use Dox-induced HCT116 cell senescence as another cellular senescent model in this study and referred to it as therapy-induced senescence (TIS). TIS is an important mechanism contributing to the effectiveness of DNA damage-based chemotherapy [23, 24]. We also used human lung fibroblast 2BS cells as a replicative senescence model.

**Figure 1:**
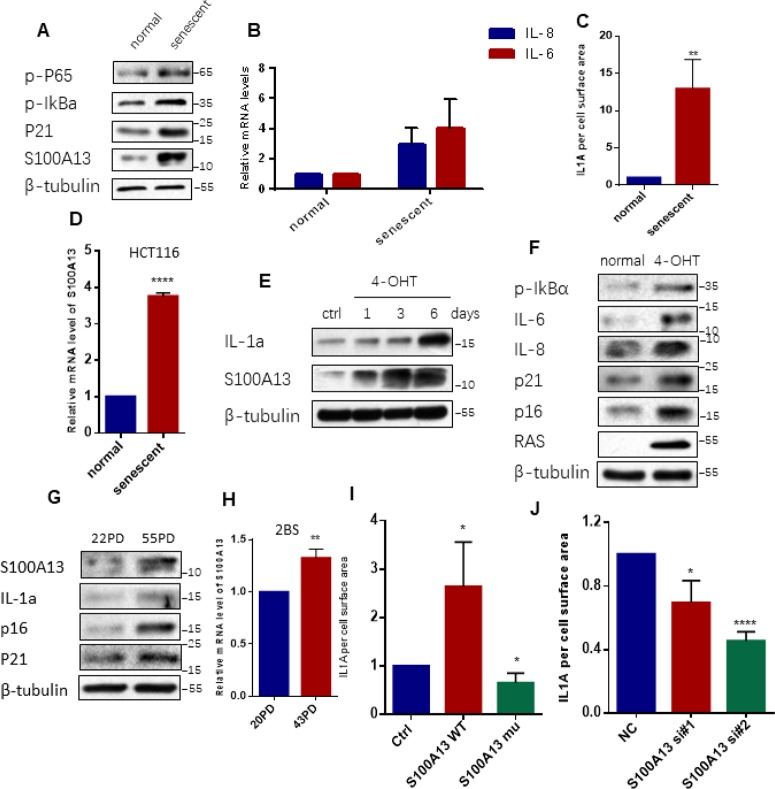
**S100A13 is up-regulated and modulates cell surface-bound IL-1α level during cellular senescence.** (**A**–**D**) Normal HCT116 cells either untreated or treated with 100 nM Dox for 4 days. (**A**) Cell lysates were subjected to western blot analysis using the indicated antibodies. (**B**) The mRNA levels of IL-6/IL-8 were analyzed by real-time qPCR. (**C**) The cells were collected, washed, incubated in PBS with FITC-labeled monoclonal antibodies against IL-1α, and processed by FACS analysis to determine the amount of cell surface-bound IL-1α. The histograms were the percent of total fluorescence signal subtracting the background fluorescence for unlabeled cells (n=3). (**D**) The mRNA levels of S100A13 were analyzed by real-time qPCR (n=3). (**E**) ER:Ras IMR90 cells were given 100 nM 4-OHT for the indicated days to induce Ras expression, fresh medium with 4-OHT was changed every other day. Cell lysates were then analyzed for expression of the indicated proteins. (**F**) ER:Ras IMR90 cells were given 4-OHT for 6 days to induce senescence. Cell lysates were then subjected to Western blot analysis for the indicated proteins. (**G** and **H**) 20PD, 55PD and 43PD 2BS cells were collected, and total proteins and/or total RNAs were extracted, respectively. (**G**) Cell lysates were analyzed by Western blot for the indicated proteins. (**H**) The mRNA levels of S100A13 were analyzed by real-time qPCR (n=3). (**I**) Cells transfected with the siRNA#2 against S100A13 first, then followed by transfection with control vector, the same sense mutation plasmids of S100A13 wild type or mutant type which were insensitive to the siRNA#2 , and then treated with Dox (100 nM) for 3 days, and then cell surface-bound IL-1α were analyzed by FACS (n=3). (**J**) Cells transfected with the control (siNC) or two independent siRNAs against S100A13, then treated with Dox (100 nM) for 4 days, and then cell surface-bound IL-1α were analyzed by FACS (n=3). Three independent experiments were analyzed. Error bars represent means ± SD (n = 3) *P < 0.05, **P < 0.01, ***P < 0.005, ****P < 0.001 in (B), (C), (D), (H), (I) and (J).

Agreed with previous report [22], chronically treated HCT116 cells with low dose of Dox induced NF-κB activation and subsequent IL-6/IL-8 mRNAs production, as well as p21 up-regulation when compared to the untreated cells (Figure 1A, 1B), which indicated that HCT116 cells underwent senescence and developed SASP. The SA-β-gal staining result also confirmed the senescent state of HCT116 cells (Supplementary Figure 1A and 1B). Consistent with published data [11], cell surface-associated IL-1α which detected by FACS-based measurement by using a fluorescent-labeled anti-IL-1α monoclonal antibody, largely increased in senescent HCT116 cells relative to normal HCT116 cells (Figure 1C, and Supplementary Figure 1C). Importantly, S100A13, a core factor in mediating the non-classical secretion of IL-1α, was dramatically up-regulated in senescent HCT116 cells compared to normal HCT116 cells (Figure 1A). Furthermore, the mRNA level of S100A13 in TIS cells increased almost four times relative to normal cells (Figure 1D).

In Ras OIS model, we determined the kinetics of S100A13 and IL-1α expressions upon Ras induction. In the time-course assay, we found IL-1α expression levels gradually increased with Ras induction, peaking at day 6 when cells entered senescent phase. Meanwhile, S100A13 levels also gradually elevated and reached to the peak level at day 6, which correlated very well with IL-1α expression levels (Figure 1E). The senescent state of ER:Ras-IMR90 cells was characterized by IκBα phosphorylation, IL-6/IL-8 elevation, as well as p16 and p21 up-regulation with Ras induction (Figure 1F).

We further evaluated S100A13 expression levels in 20 population doublings (PD, young) and 55PD (senescent) 2BS cells. Similar to TIS and Ras OIS results, S100A13 was also markedly induced in 55PD 2BS cells relative to 20PD 2BS cells. Meanwhile, IL-1α expression was also elevated. In addition, p16 INK4a and p21 levels substantially increased following replicative cellular senescence (Figure 1G). Moreover, S100A13 mRNA level in 43PD cells was also significantly higher than in 20PD cells (Figure 1H).

We then investigated whether S100A13 regulated non-classical secretion of IL-1α during cellular senescence. To eliminate the up-regulation of endogenous S100A13 during cellular senescence, we first depleted S100A13 using siRNA in normal HCT116 cells, then overexpressed siRNA-insensitive wild type S100A13 and induced cells to senescence. FACS analysis of cell surface-bound IL-1α showed that S100A13 overexpression significantly enhanced IL-1α levels compared to empty vector control cells (Figure 1I, and Supplementary Figure 1C). The basic residue (BR)-rich domain at the C-terminus of S100A13 is novel among the various members of the S100 gene family [25], and a dominant-negative S100A13 mutant lacking the last eleven residues in this domain has been shown to lose the ability to associate with IL-1α and then impair the secretion of IL-1α [21]. Therefore, we generated siRNA-insensitive dominant-negative S100A13 mutant and overexpressed it in HCT116 cells. Overexpression of S100A13 mutant significantly reduced the levels of cell surface-bound IL-1α relative to the vector control TIS cells (Figure 1I, and Supplementary Figure 1C). Similarly, S100A13 knockdown with two independent siRNAs also significantly decreased cell surface-bound IL-1α levels (Figure 1J, and Supplementary Figure 1D). These results demonstrate that S100A13 is induced and regulates the non-classical secretion of IL-1α during SASP-competent cellular senescence.

### S100A13 regulates NF-κB activity and SASP induction during cell senescence

**Figure 2:**
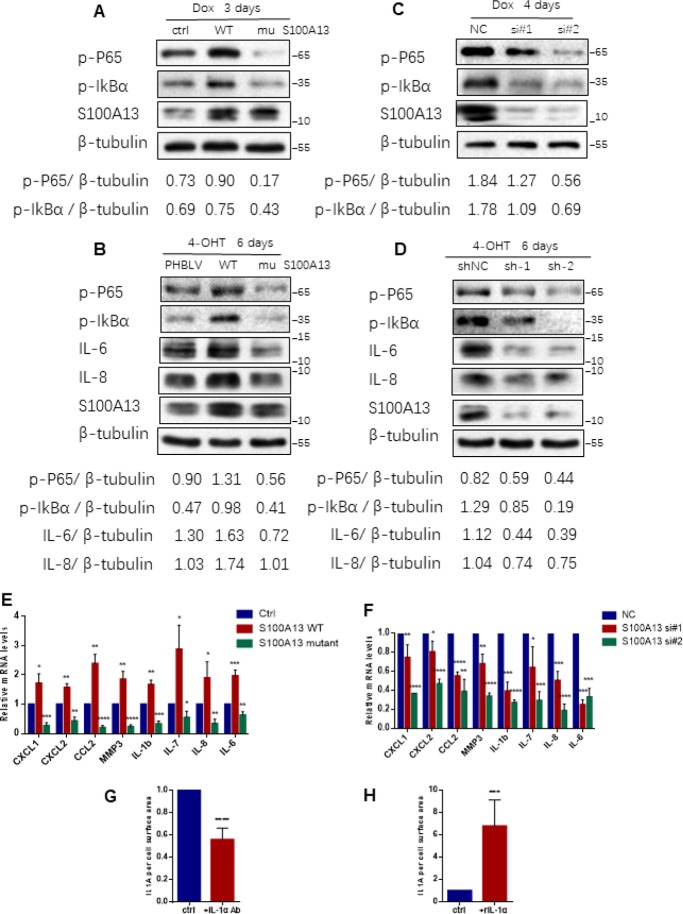
**S100A13 modulates NF-κB activity and SASP expression during cell senescence.** (**A**) HCT116 cells were transfected with the control vector, S100A13 wild type or mutant type, and treated with Dox (100 nM) for 3 days. Then cell lysates were subjected to western blot analysis using the indicated antibodies. (**B**) ER:Ras IMR90 cells stably transduced with PHBLV, S100A13 wild type or mutant type were given 4-OHT for 6 days. Cell lysates were then subjected to Western blot analysis for the indicated proteins. (**C**) HCT116 cells were transfected with control (siNC) or two independent siRNAs against S100A13, and treated with Dox (100 nM) for 4 days. Then cell lysates were subjected to western blot analysis using the indicated antibodies. (**D**) ER:Ras IMR90 cells stably transduced with control (siNC) or two independent shRNAs against S100A13, were given 4-OHT for 6 days. Cell lysates were then subjected to Western blot analysis for the indicated proteins. (**E** and **F**) HCT116 cells were transfected with vector, S100A13 wild type or mutant type, or transfected with control (siNC) or two independent siRNAs against S100A13, and treated with Dox (100 nM) for 4 days. Then mRNA levels of some SASP genes were analyzed by real-time qPCR (n=3). (**G**) HCT116 cells were transfected with wild type S100A13, and treated with Dox (100 nM) for 3 days. Control IgG (0.6 ug/ml) or neutralizing antibody IL-1a (0.6 ug/ml) were added for the last 2 days. Then cell surface-bound IL-1α were analyzed by FACS (n=3). (**H**) HCT116 cells were transfected with siRNA#2 against S100A13, and treated with Dox (100 nM) for 3 days. Solvent or recombinant IL-1a protein (300 ng/ml) were added for the last 2 days. Then cell surface-bound IL-1α were analyzed by FACS (n=3). Three independent experiments were performed. Error bars represent means ± SD (n = 3) *P < 0.05, **P < 0.01, ***P < 0.005, ****P < 0.001 in (E–H).

NF-κB is a downstream target of IL-1R signaling activated by cell surface-bound IL-1α in various types of cell senescence [11], thus we next examined whether S100A13 regulated NF-κB activity through modulation of IL-1α secretion during cell senescence. For this purpose, we genetically overexpressed, mutated or silenced S100A13 in HCT116 cells and ER:Ras IMR90 cells and then induced them to senescence, respectively. S100A13 overexpression considerably promoted p65 RelA and IκBα phosphorylation levels when compared with the vector control cells both during the induction of TIS and Ras OIS (Figure 2A, 2B, and Supplementary Figure 2A, 2B). In contrast, S100A13 mutant markedly suppressed p65 RelA and IκBα phosphorylation levels relative to the vector control cells both during the induction of TIS and OIS (Figure 2A, 2B, and Supplementary Figure 2A, 2B). Similar to S100A13 mutant results, S100A13 depletion with two independent siRNAs or shRNAs also dramatically repressed p65 RelA and IκBα phosphorylation levels compared to control cells both during TIS and Ras OIS (Figure 2C, 2D, and Supplementary Figure 2C, 2D). Given that NF-κB is a master regulator of many SASP genes transcription and S100A13 modulates NF-κB activity, we used quantitative RT-PCR to determine mRNA levels of multiple SASP genes which might be affected by S100A13 during cellular senescence. Compared to control cells, S100A13 overexpression increased chemokines such as CXCL1, CXCL2, and CCL2, and matrix metalloproteinase MMP3, as well as pro-inflammatory cytokines including IL-1β, IL-6, IL-7, and IL-8 mRNA levels, whereas S100A13 mutant alleviated these SASP genes transcription during the induction of TIS (Figure 2E). Furthermore, loss of S100A13 markedly attenuated these SASP genes mRNA levels during the induction of TIS (Figure 2F). Similar to TIS results, S100A13 overexpression augmented IL-6 and IL-8 expression levels, while S100A13 mutant or knockdown significantly reduced IL-6 and IL-8 expression levels during Ras OIS (Figure 2B, 2D).

We further validated whether S100A13 regulated NF-κB activity and SASP genes expressions during cellular senescence depending on the modulation of cell surface-bound IL-1α levels. In this regard, first we used IL-1α monoclonal antibody to block interaction between IL-1α and IL-1R [11]. S100A13-overexpressed HCT116 cells were induced to senescence by Doxorubicin, and then IL-1α neutralizing antibody was added to cells for the last 2 days. Compared with control cells, IL-1α antibody substantially reduced cell surface-bound IL-1α level (Figure 2G), p65 RelA and IκBα phosphorylation levels (Supplementary Figure 3A), and multiple SASP genes mRNA levels (Supplementary Figure 3B). Next, we added recombinant IL-1α (rIL-1α) to S100A13-depleted HCT116 cells in the presence of Dox treatment, which expressed low levels of cell surface-bound IL-1α. rIL-1α dramatically increased cell surface-bound IL-1α level (Figure 2H), p65 RelA and IκBα phosphorylation levels (Supplementary Figure 3C), and many SASP genes mRNA abundances (Supplementary Figure 3D), when compared with control cells. Therefore, the inhibition of NF-κB activity and SASP genes expressions in S100A13-depleted cells could be rescued by adding rIL-1α. Based on these evidences, we conclude that S100A13 regulates the initiation of NF-κB activity and subsequent SASP genes inductions via modulation of the non-classical secretion of IL-1α during cellular senescent process.

### Blockage of IL-1α binding to the S100A13 or to the Cu^2+ ^inhibits cell surface-bound IL-1α, NF-κB activity, and SASP induction

**Figure 3:**
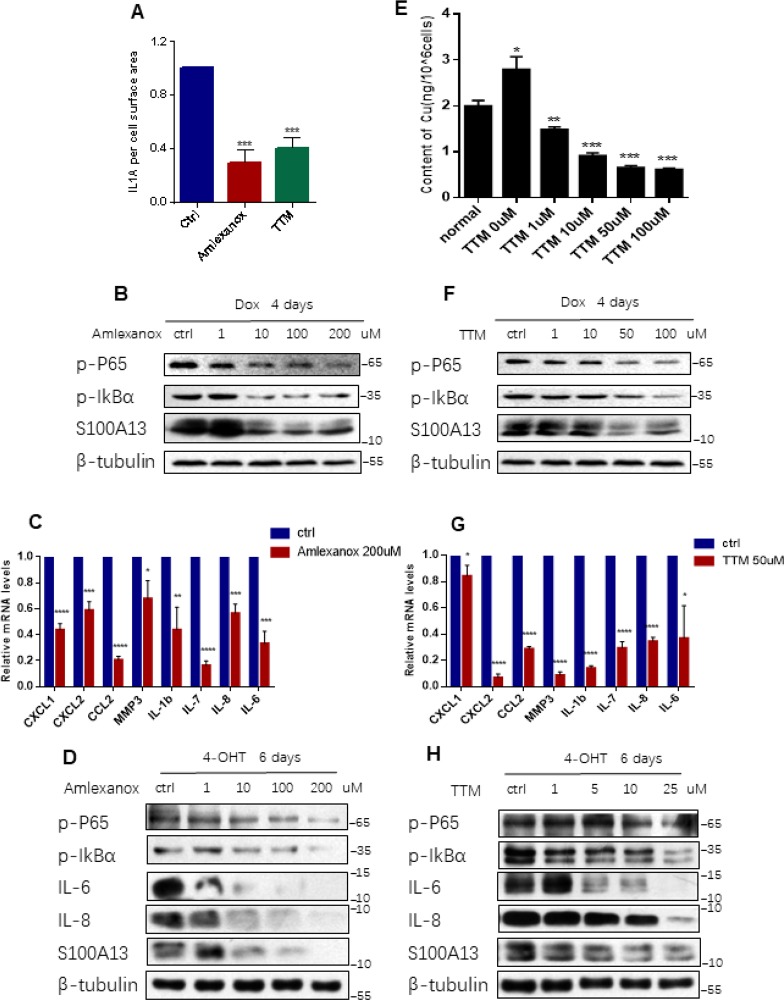
**Inhibition the binding of IL-1α to S100A13 or to Cu^2+ ^suppresses IL-1α secretion, NF-κB activity, and SASP expression.** (**A**–**C**) HCT116 cells were treated with Dox (100 nM) for 4 days in the presence of the indicated doses of Amlexanox or TTM. Then, (**A**) Cell surface-bound IL-1α were analyzed by FACS (n=3). (**B**) Cell lysates were subjected to western blot analysis for the indicated proteins. (**C**) mRNA levels of some SASP genes were analyzed by real-time qPCR (n=3). (**D**) ER:Ras IMR90 cells were given 4-OHT for toal 6 days in the presence of the indicated doses of Amlexanox; fresh medium with 4-OHT and Amlexanox was changed every other day. Cell lysates were then subjected to Western blot analysis for the indicated proteins. (**E–G**) HCT116 cells were treated without or with Dox (100 nM) and the indicated doses of TTM for 4 days. Then, cells were collected, washed with PBS for twice, and, (**E**) Analyzed the intracellular Cu^2+^ concentration by ICP-MS (n=3). (**F**) Cell lysates were subjected to western blot analysis for the indicated proteins. (**G**) mRNA levels of some SASP genes were analyzed by real-time qPCR (n=3). (**H**) ER:Ras IMR90 cells were treated with TTM as in (D), then the indicated proteins were analyzed by Western blot. Three independent experiments were performed and analyzed. Error bars represent means ± SD (n = 3) *P < 0.05, **P < 0.01, ***P < 0.005, ****P < 0.001 in (A), (C), (E), and (G).

The association of IL-1α with S100A13 is the key step in the non-classical secretion of IL-1α [19]. Amlexanox is a drug known to inhibit the binding of IL-1α to the S100A13 thus disrupting the formation of S100A13-IL-1α heterotetrameric complex [19]. There is also a direct experimental evidence to show that Amlexanox inhibits the secretion of IL-1α [26], which is similar to that reported for the inhibition of FGF1 release [27]. To further probe the role of the non-classical secretory pathway of IL-1α in modulation of NF-κB activity and SASP genes expressions during cellular senescence, we treated HCT116 cells with Dox to induce cell senescence and added Amlexanox simultaneously. Prior to investigating the effect of Amlexanox, CCK-8 assay was performed to determine the cytotoxicity of Amlexanox to HCT116 cells. The cell viability result showed that about 20% cells were death at 200 uM dose of Amlexanox treatment, therefore we exposed HCT116 cells to the Amlexanox no more than 200 uM (Supplementary Figure 4A). Amlexanox treatment greatly blunted the levels of cell surface-bound IL-1α (Figure 3A, and Supplementary Figure 4B), inhibited p65 RelA and IκBα phosphorylation levels, as well as S100A13 inductions, in a dose-dependent manner, and significantly suppressed multiple SASP genes mRNA levels during the induction of TIS (Figure 3B and 3C), which was similar to the effects of S100A13 mutant or S100A13 knockdown on NF-κB activity and SASP genes expressions during the induction of TIS. We also treated IMR90 cells with Amlexanox in the presence of Ras induction. Consistent with TIS results, Amlexanox treatment dose-dependently inhibited p65 RelA and IκBα phosphorylation levels, as well as S100A13 expressions, during the induction of Ras OIS (Figure 3D). Amlexanox treatment also dose-dependently inhibited IL-6 and IL-8 protein levels during the induction of Ras OIS (Figure 3D), which agreed with Amlexanox repressed SASP genes mRNA levels (Figure 3C).These results further support the notion that the non-classical secretory pathway of IL-1α is important for the stimulation of NF-κB activity and subsequent SASP genes inductions.

It is reported that IL-1α is a Cu^2+^-binding protein and the intracellular Cu^2+^ ions are required for the export of IL-1α to the extracellular membrane [21]. To probe whether Cu^2+^ could regulate IL-1α secretion, NF-κB activity and SASP genes expressions during cellular senescence, we used Cu^2+^ chelator, tetrathiomolybdate (TTM) to treat HCT116 cells [28]. CCK-8 assay result showed that cell death ratio was less than 20% when treated normal HCT116 cells with 100 uM TTM (Supplementary Figure 4C). First, we used inductively coupled plasma mass spectrometry (ICP-MS) to detect intracellular Cu^2+ ^concentrations in normal and TIS HCT116 cells, respectively. As shown in Figure 3E, Cu^2+ ^concentration in TIS cells was significant higher than that in the normal cells, and TTM treatment reduced intracellular Cu^2+^ concentrations in a dose dependent manner during the induction of TIS. Then we exploited the effect of TTM on IL-1α secretion, NF-κB activity and SASP genes expressions during TIS. TTM treatment remarkably repressed cell surface-bound IL-1α levels (Figure 3A, and Supplementary Figure 4B), dose-dependently inhibited p65 RelA and IκBα phosphorylation levels as well as S100A13 inductions (Figure 3F), and notably restrained multiple SASP genes expressions when compared with the untreated control cells during the induction of TIS (Figure 3G). The similar prohibition of p65 RelA and IκBα phosphorylation levels as well as S100A13 expressions was obtained when treated IMR90 cells with TTM during Ras OIS (Figure 3H). TTM treatment also dose-dependently inhibited IL-6 and IL-8 protein levels during the induction of Ras OIS (Figure 3H), which was consistent with TTM suppressed SASP genes mRNA levels in TIS (Figure 3G). These results imply that Cu^2+^ takes part in the regulation of NF-κB activity and subsequent SASP genes expressions via modulation of the non-classical secretion of IL-1α.

### S100A13 regulates Ras OIS and TIS responses

**Figure 4:**
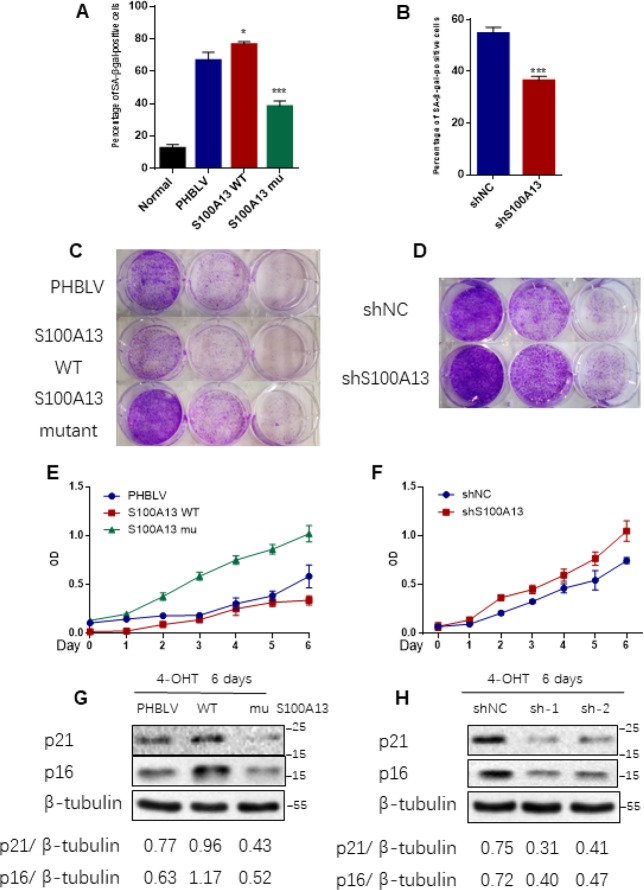
**S100A13 overexpression enhances Ras OIS, while S100A13 silencing attenuates Ras OIS.** (**A** and **B**) Normal IMR90 cells were infected with lentivirus carrying oncogenic RAS to induce OIS, and at the same time cells were co-infected with (**A**) PHBLV, S100A13 wild type or mutant type, or (**B**) control vector (shNC), shRNA against S100A13. After 6 days, cells were stained for SA-β-gal activity. The percentages of cells positive for SA-β-gal were calculated and graphed (n=3). Error bars represent means ± SD from three independent experiments. *P < 0.05, **P < 0.01, ***P < 0.005. (**C** and **D**) IMR90 cells were infected as (A) and (B) and cultured for 10–14 days. Then, colony formation assay was performed. (**E** and **F**) IMR90 cells were infected as (A) and (B), and cell growth curves were determined by CCK-8/WST-8 assay for the indicated time. Values represent the means ± SD of triplicate points from a representative experiment (n = 3), which was repeated three times with similar results. (**G** and **H**) ER:Ras IMR90 cells were infected as (A) and (B), and were given 4-OHT for 6 days. Then, the indicated proteins were detected by Western blot analysis.

Given that SASP core factors IL-6/IL-8 can reinforce cell senescence and disruption of IL-6/IL-8 autocrine network alleviates Ras OIS entry or maintenance [2, 8], the results that interruption of S100A13-mediated non-classical secretion of IL-1α significantly repressed NF-κB activity and SASP inductions including IL-6/IL-8 prompted us to further decipher the role of S100A13 in the establishment of Ras OIS. To analyze the role of S100A13 in Ras OIS, we stably overexpressed wild type or mutant of S100A13, or silenced it, and co-infected with RAS in young IMR90 cells. The co-expression of S100A13, S100A13 mutant and Ras, as well as the knockdown of S100A13, were confirmed by Western blot (Supplementary Figure 5A). After 6 days Ras expression, cells were subjected to SA-β-gal activity assay, colony formation assay, and cell proliferation assay, respectively, to evaluate cellular senescent state. Enforced expression of Ras in PHBLV empty vector or shRNA empty vector-expressing cells induced senescent phenotypes which characterized by the dramatic increases of SA-β-gal activity (Figure 4A, 4B, and Supplementary Figure 5B, 5C), a biomarker for senescent cells, when compared with the control cells without Ras expression. S100A13-overexpressing cells displayed the significant higher level of SA-β-gal activity (Figure 4A, and Supplementary Figure 5B), the less number of colonies measured by colony-formation assay (Figure 4C), and more lower cell proliferation rate (Figure 4E) when compared with PHBLV vector control cells, which indicated that S100A13 overexpression induced more severe senescent phenotypes than vector control cells in the presence of Ras induction. Conversely, with Ras induction, S100A13 mutant or S100A13 knockdown significantly reduced SA-β-gal positive staining cells (Figure 4A, 4B, and Supplementary Figure 5B, 5C), increased colony numbers (Figure 4C, 4D), and promoted cell proliferation (Figure 4E, 4F), when compared with vector control cells, which suggested that Ras OIS was notably prevented. Moreover, S100A13 overexpression elevated the levels of cell cycle inhibitors p16 and p21, two well-known cell senescence markers, whereas S100A13 mutant or S100A13 knockdown greatly repressed p16 and p21 inductions during Ras OIS (Figure 4G, 4H). Similarly, S100A13 overexpression augmented, while S100A13 mutant or S100A13 silencing decreased p21 levels, respectively, during TIS in HCT116 cells (Supplementary Figure 5D–5F).

We further used Amlexanox and TTM to examine the role of the non-classical secretory pathway of IL-1α on the establishment of Ras OIS and TIS. Amlexanox or TTM treatment also dramatically reduced the SA-β-gal activity in Ras-expressed IMR90 cells, and dose-dependently depressed p16 and p21 expressions during Ras OIS and TIS (Supplementary Figure 6A–6F). Collectively, these results suggest that S100A13 and its-mediated non-classical secretory pathway of IL-1α play a critical role in regulating the establishment of Ras OIS and TIS. Augment of S100A13 promotes cellular senescent response, while impairment of S100A13-mediated non-classical secretory pathway of IL-1α at least largely postpones cellular senescent response.

### S100A13 regulates replicative cellular senescent process

**Figure 5:**
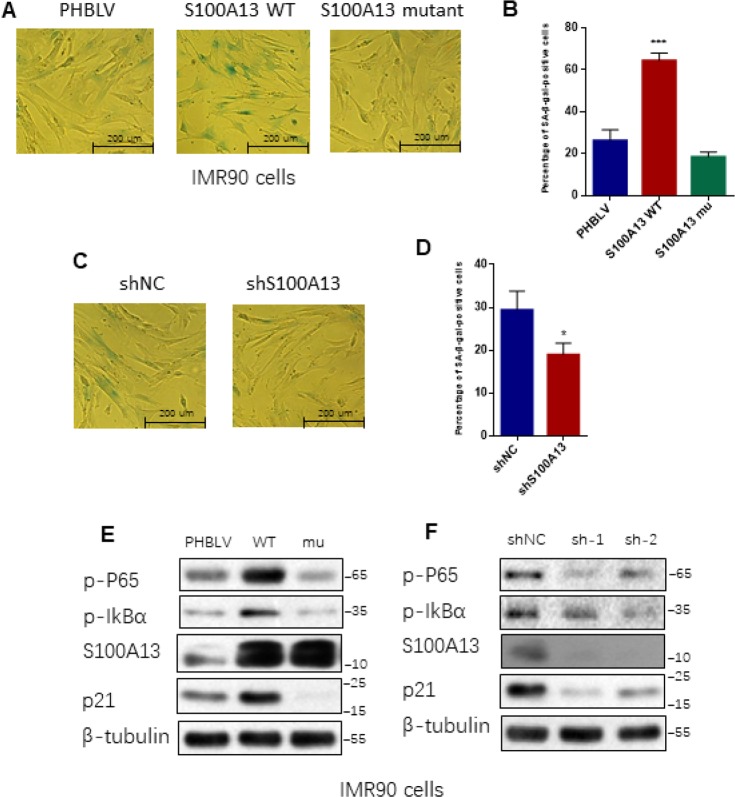
**S100A13 overexpression promotes, whereas S100A13 knockdown delays replicative cellular senescence in IMR90 cells.** (**A**–**D**) 36PD normal IMR90 cells were infected with (**A**) PHBLV, wild type S100A13, S100A13 mutant, or (**C**) shRNA empty vector (shNC), shRNA#2 against S100A13, respectively, then cultured the infected-cells to 40PD. Cells were then stained for SA-β-gal activity (**A** and **C**). The percentages of cells positive for SA-β-gal were calculated and graphed (**B** and **D**) (n=3). Error bars represent means ± SD from three independent experiments. *P < 0.05, **P < 0.01, ***P < 0.005. (**E** and **F**) The indicated proteins extracted from above samples were detected by western blot.

We further determined the functional role of S100A13 in replicative cellular senescence. We stably overexpressed wild type or mutant of S100A13, or silenced S100A13 in middle-aged IMR90 cells (36PD) and cultured them to 40PD, and then examined their effects on the replicative cellular senescent process. S100A13-overexpressing cells possessed at least two fold increases of SA-β-gal activity compared to corresponding mock cells after 4 cell passages (Figure 5A, 5B). In contrast, S100A13 mutant significantly decreased the percentage of SA-β-gal positive staining cells (Figure 5A, 5B). Similar to S100A13 mutant result, loss of S100A13 also significantly repressed SA-β-gal activity compared to corresponding mock cells after 4 cell passages (Figure 5C, 5D). We also tested whether S100A13 could affect NF-κB activity and other cellular senescent markers in these IMR90 cells. S100A13 overexpression notably enhanced p65 RelA and IκBα phosphorylation levels when compared with the vector control cells after 4 cell passages, whereas S100A13 mutant or S100A13 silencing considerably restrained p65 RelA and IκBα phosphorylation levels relative to the vector control cells in 40 PD IMR90 cells (Figure 5E, 5F). In addition, S100A13 overexpression augmented, while S100A13 mutant or S100A13 silencing decreased p21 levels, respectively, during replicative cellular senescence (Figure 5E, 5F). These results were consistent with the effect of S100A13 on Ras OIS and TIS response. Taken together, these results exhibit that S100A13 and its-mediated non-classical secretory pathway of IL-1α are essential for various types of SASP-competent cellular senescent responses, including replicative cellular senescence, Ras OIS and TIS. S100A13 overexpression accelerates cellular senescent responses, while the deficiency of this pathway at least greatly delays cellular senescent responses.

### S100A13 affects multiple SASP and cellular senescence regulators

**Figure 6:**
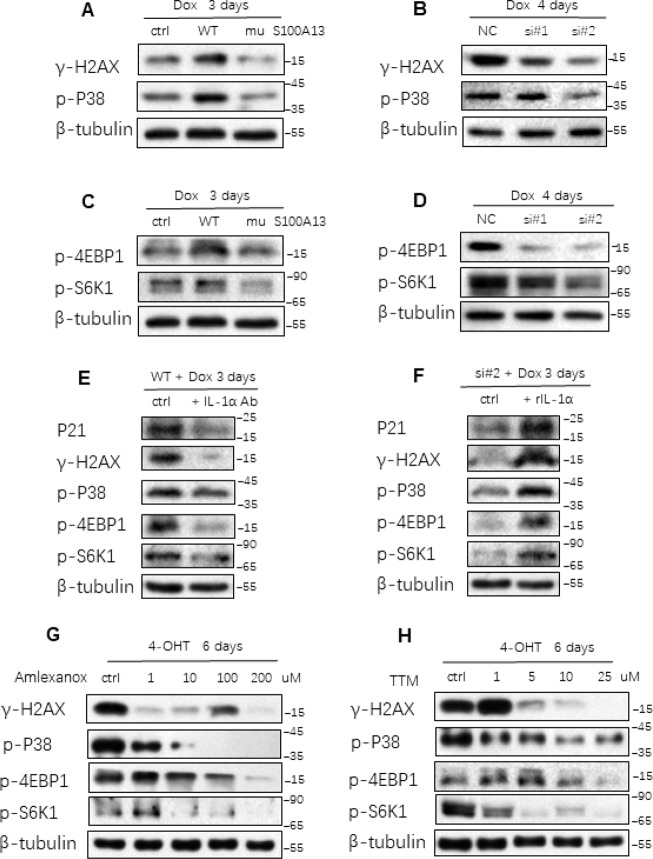
**S100A13 modulates multiple SASP and cellular senescence regulators.** (**A**–**D**) HCT116 cells were transfected with the control, S100A13 wild type or mutant type (**A** and **C**), or transfected with the control (siNC) or two independent siRNAs against S100A13 (**B** and **D**), and then treated with Dox (100 nM) for 3 or 4 days. Then cell lysates were subjected to western blot analysis for the indicated proteins. (**E**) HCT116 cells were transfected with wild type S100A13, and treated with Dox (100 nM) for 3 days. Control IgG (0.6 ug/ml) or neutralizing antibody IL-1a (0.6 ug/ml) were added for the last 2 days. Then the indicated proteins were analyzed by Western blot. (**F**) HCT116 cells were transfected with siRNA#2 against S100A13, and treated with Dox (100 nM) for 3 days. Solvent or recombinant IL-1a protein (300 ng/ml) were added for the last 2 days. Then the indicated proteins were analyzed by Western blot. (**G** and **H**) ER:Ras IMR90 cells were given 4-OHT for toal 6 days in the presence of the indicated doses of Amlexanox (**G**); or TTM (**H**); then the indicated proteins were analyzed by Western blot. Three independent experiments were performed.

Multiple signaling pathways are implicated in mediating SASP production and triggering cellular senescent response. Persistent DDR pathway is activated in various types of cell senescence and plays a critical role in inducing NF-κB activity and SASP expression as well as cellular senescent response [9]. p38 MAPK activity is up-regulated in cell senescence and is essential for the NF-κB activity and SASP induction independent of persistent DDR pathway [10]. On the other hand, reducing NF-κB/p65 activity in turn attenuates DNA damage and stress responses thus mitigating cellular senescence [29]. Considering S100A13 could regulate NF-κB activity and SASP expression by altering cell surface-associated IL-1α level, we further explored whether S100A13 could affect these cellular senescence and SASP regulators. As shown in Figure 6A, S100A13 overexpression notably enhanced γ-H2AX levels during TIS compared to vector control cells, which indicated stronger DDR, whereas S100A13 mutant largely reduced γ-H2AX levels, which implied the lower DDR level. Similar to S100A13 mutant result, knockdown of S100A13 also significantly alleviated γ-H2AX levels (Figure 6B). Likewise, S100A13 increased p38 MAPK phosphorylation level, while S100A13 mutant or depletion decreased p38 activity during the induction of TIS (Figure 6A and 6B). Altering S100A13 protein levels also affected p38 activity during Ras OIS, which was similar to TIS results (Supplementary Figure 7A, 7B).

mTORC1 activity is induced during cellular senescence and regulates many SASP mRNAs translations, especially for IL-1α translation, through phosphorylation of mTORC1 downstream targets S6K1 and 4EBP1 [30, 31]. Therefore, we further investigated the effect of S100A13 on mTORC1 activity during cellular senescence. As shown in Figure 6C, S100A13 overexpression substantially enhanced the phosphorylation levels of S6K1 and 4EBP1, which indicated the promotion of SASP transcripts translations during the induction of TIS. In contrast, S100A13 mutant or S100A13 silencing considerably suppressed the phosphorylation levels of S6K1 and 4EBP1, which suggested the inhibition of SASP mRNAs translations during the induction of TIS (Figure 6C and 6D, and Supplementary Figure 7C, 7D). S100A13 also affected mTORC1 activity during the induction of Ras OIS (Supplementary Figure 7A, 7B). Altogether, these results suggest that S100A13 is able to affect multiple SASP and cellular senescence regulators.

We further examined whether IL-1α was required for S100A13 to modulate these cellular senescence regulators. IL-1α antibody drastically reduced γ-H2AX, p-p38, p-S6K1 and p-4EBP1 levels as well as p21 expressions in S100A13-overexpressed HCT116 cells during TIS when compared with untreated control cells (Figure 6E). Likewise, rIL-1α remarkably promoted γ-H2AX, p-p38, p-S6K1, p-4EBP1, and p21 levels in S100A13-depleted HCT116 cells during TIS relative to untreated control cells (Figure 6F). These results indicate that S100A13 regulates cellular senescent process depending on the modulation of cell surface-bound IL-1α level.

Last, we used Amlexanox and TTM to examine the effect of the non-classical secretory pathway of IL-1α on these regulators during Ras OIS and TIS. Amlexanox and TTM treatments dose-dependently inhibited γ-H2AX levels, and diminished p-p38, p-S6K1 as well as p-4EBP1 levels during both Ras OIS and TIS (Figure 6G, 6H, and Supplementary Figure 7E, 7F). Collectively, these results demonstrate that inhibition of the non-classical secretory pathway of IL-1α alleviates the inductions of multiple SASP and cellular senescence regulators.

## DISCUSSION

IL-1α is a pro-inflammatory cytokine and plays a key role in establishing and maintaining the SASP through comprising a positive feedback loop with NF-κB that ultimately induces many SASP genes transcriptions [11]. SASP-competent senescent cells express high levels of IL-1α mRNA, intracellular protein, and cell surface-bound IL-1α, but secret very little IL-1α to extracellular compartment. Since devoid of an N-terminus signal peptide, IL-1α needs to combine with S100A13 and Cu^2+^ to form complex first, then secrets to cell membrane surface through the non-classical secretory pathway [19–21]. Although S100A13 and Cu^2+^ are critical for IL-1α release to cell surface, however, whether the non-classical secretory pathway of IL-1α plays a role in SASP expression and cellular senescence remains unknown. In this study, we found that S100A13 and Cu^2+^ levels were up-regulated in various types of cellular senescence and involved in regulating the abundance of cell surface-associated IL-1α, NF-κB activity, SASP induction, and ultimately cellular senescent response (Figures 1–6). Thus, we uncovered a previously unidentified connection between non-classical secretory pathway of IL-1α and SASP expression as well as cell senescence.

Various cellular stresses such as DNA damage, oxidative stress, and oncogenes activation induce cell senescence and lead to NF-κB activation and subsequent SASP expression [1]. Though the activation of NF-κB and subsequent SASP induction reinforce cell senescence, this in turn drives even more cellular damage. Conversely, inhibition of NF-κB activity attenuates multiple cellular senescent regulators and markers, including γ-H2AX, SA-β-gal activity, and p16, etc [29]. Agreement with this notion, we found that S100A13 overexpression enhanced NF-κB activity and SASP genes transcriptions (Figure 2A, 2B, and 2E), promoted Ras OIS, TIS, and replicative cellular senescent responses, which was accompanied with the up-regulation of cellular senescent markers such as p16 and p21 (Figure 4, 5, and Supplementary Figure 5). These phenotypes correlated well with the augmentation of many cellular senescence and SASP regulators including γ-H2AX, p38, and mTORC1 (Figure 6A, 6C, and Supplementary Figure 7A, 7C) during cellular senescent process. In contrast, either S100A13 mutant, or S100A13 silencing, or disruption of non-classical secretory pathway of IL-1α by Amlexanox or TTM, all of these attenuated NF-κB activity (Figure 2A–2D, and Figure 3B, 3D, 3F, 3H), which resulted in the decrease of SASP genes transcriptions (Figure 2E, 2F, and Figure 3C, 3G), and the delay of Ras OIS, TIS, and replicative cellular senescent responses, as well as the reductions of cellular senescent markers p16 and p21 (Figure 4, 5, and Supplementary Figure 5, 6). These phenotypes correlated with the down-regulation of cellular senescence and SASP regulators γ-H2AX, p38, and mTORC1 (Figure 6, and Supplementary Figure 7) during cellular senescent process. We also proved that S100A13 could regulate cellular senescent process all depending on its capacity to modulate cell surface-bound IL-1α levels (Figure 1I, 1J, 2G, 2H, 6E, 6F, and Supplementary Figure 3). Therefore, S100A13 and its-mediated non-classical secretory pathway of IL-1α, may represent as potential targets to delay cellular aging.

S100A13 is a member of a large gene family of small acidic proteins characterized by the absence of a classical signal peptide sequence and the presence of two Ca^2+^-binding EF-hand domains [18]. The well-known function of S100A13 is mediating the secretion of some proteins such as FGF-1 and IL-1α via the non-classical secretory pathway [20]. However, whether S100A13 has a role in SASP induction and cell senescence has never been exploited. In this study, we showed that S100A13 protein level was up-regulated during cellular senescence, and S100A13 increased the cell surface-bound IL-1α levels, then stimulated NF-κB activity and subsequent SASP induction, and ultimately promoted cellular senescent response. Conversely, S100A13 knockdown attenuated cellular senescent responses (Figures 1, 2, 4–6). To our knowledge, this is the first time to unveil the role of S100A13 in modulating SASP production and cell senescence. We further found that the mRNA levels of S100A13 were up-regulated in variety of cellular senescence (Figure 1D, 1H), which indicated that S100A13 at least partly was regulated at transcriptional level by currently undefined transcription factor or subjected to post-transcriptional level modification. It needs to be investigated in the future. Therefore, S100A13 induction may be a universal response in the SASP-producing cellular senescent process. S100A13 has also been implicated in the regulation of some cancer. The up-regulation of S100A13 is found in thyroid cancer [32], malignant melanoma [33], and lung cancer [34], etc., which is associated with promoting cancer cell proliferation, invasion, and poor prognosis. However, in our study, we demonstrated that S100A13 knockdown led to alleviate Ras OIS (Figure 4). Our findings suggest that S100A13 may potentially have tumor suppressive function at an early stage of cancer development.

Copper (Cu) is an essential trace element that acts as a co-factor of different enzymes such as cytochrome c oxidase, Cu/Zn superoxide dismutase, and others [35]. Cu ions have been implicated in the various age-associated neurodegenerative disorders, such as Wilson’s disease, Alzheimer’s, and Parkinson’s disease [36], and other pathologies including cancer [37]. Recently, it is reported that intracellular Cu^2+^ level is elevated during cellular replicative senescence and sublethal concentration of Cu^2+^ could induce cell premature senescence [38]. Consistent with this finding, our result also showed that the intracellular Cu^2+^ level was up-regulated during cell senescence (Figure 3E). Meanwhile, we unraveled that Cu^2+^ was required for the non-classical secretion of IL-1α, NF-κB activation and subsequent SASP expression and cellular senescent response (Figure 3F–3H, 6H, and Supplementary Figure 4B, 6, and 7F). Both ROS signaling and p38 MAPK partly mediate Cu^2+^-induced cell senescence [38], and it is known that both ROS signaling and p38 MAPK are important regulators of SASP induction [10, 39]. Our results showed that manipulation of non-classical secretory pathway of IL-1α affected p38 activity. Therefore, we speculate that multiple mechanisms may involve in mediating Cu^2+^-induced cell senescence, including ROS, p38 MAPK, and SASP.

TTM is a pharmacological agent which can chelate with Cu^2+^ and used to treat Wilson’s disease, a genetic disorder of excess Cu^2+^ accumulation [28]. In addition, TTM is being used in clinical trials for the treatment of several human tumors as a potential anti-angiogenic agent [40]. However, whether TTM has potential effect on cell senescence has never been reported. Amlexanox is a drug known to inhibit the binding of IL-1α to the S100A13 thus impairing the secretion of IL-1α [19, 26]. We showed here that both TTM and Amlexanox were able to dampen SASP productions via disruption of IL-1α secretion and NF-κB activity (Figure 3, 6G, 6H, and Supplementary Figure 4, 6, and 7E, 7F). Hence, TTM and Amlexanox can be the potential inhibitors of SASP production. SASP possesses tumor suppressive functions via reinforcing cell senescence and its associated immune surveillance, therefore, TTM or Amlexanox treatment may be harmful during the early stages of cancer. On the other hand, pro-inflammatory SASP factors can promote tumor growth and invasion, or contribute to the aging and age-related diseases by elevating the chronic, low-grade inflammation in aged tissues, thus SASP can be potentially detrimental to organisms in late life. Therefore, the reduction of SASP caused by TTM or Amlexanox treatment might be benefit for the organisms in late life.

In summary, we show here that S100A13 promotes cell senescence by elevating the level of cell surface-bound IL-1α thus enhancing NF-κB activity and subsequent SASP induction. S100A13 and Cu^2+ ^are essential for the induction of at least subset of SASP. Ablation of S100A13 mitigates cellular senescent response.

## MATERIALS AND METHODS

### Cell culture and senescence induction

HCT116 cells were cultured in DMEM supplemented with 10% FBS. Normal HCT116 cells were treated with 100 nM Doxorubicin for 4 days to induce senescence. IMR90 human diploid fibroblasts (HDFs) were purchased from the American Type Culture Collection (ATCC) and cultured in DMEM with 10% FBS. ER:Ras-IMR90 cells were generously provided by Masashi Narita, Cancer Research U.K., Cambridge Research Institute, and were given 100 nM 4-hydroxytamoxifen (4-OHT) to induce ER-Ras fusion protein expression and maintained in 4-OHT–containing DMEM (w/o phenol red) with 10% FBS until harvesting. Human diploid 2BS fibroblasts cells (National Institute of Biological Products, Beijing, China) were cultured in DMEM supplemented with 10% FBS.

### Antibodies and reagents

The following primary antibodies were used for immunoblotting analysis: anti-p-P65 (#3033) and anti-p-IκBα (#2859) (Cell Signaling Technology), Anti-IL-1α-Fluorescein (#IC200F, R&D Systems), Anti-p16 (#10883-1-AP, Proteintech), anti-S100A13, IL-1α, p-P38, γ-H2AX, p-4EBP1, p-S6K1, RAS, E1A (Santa Cruz), anti-p21 and anti-β-tubulin (#BS1482M) (BioWorld). IgG (Santa Cruz, sc-2025). Human recombinant IL-1α (Sino Biological, 10128-HNCH).

DMSO (Sigma) was used as a solvent to dissolve TTM (Santa Cruz) and Amlexanox (Selleck). 4-OHT (Sigma) was dissolved in methanol. Doxorubicin (Sigma) was dissolved in sterile water.

### Plasmids and transfection

Full-length S100A13 and S100A13 mutant plasmids were transiently transfected into cells with PEI Reagent. Two independent siRNA sequences against S100A13 were: siRNA#1: 5’- GCGUCAACGAGUUCAAAGATT-3’, and siRNA#2: 5’- GCUUGGAUGUGAAUCAGGATT-3’. The same sense mutation plasmids of wild type S100A13 and S100A13 mutant which were insensitive to the siRNA#2 were generated by using sequence: AAGAGCTTGGACGTAAACCAGGACTCGGAGCTCAAGTT. siRNAs were transfected using Lipofectamine RNAiMAX Reagent following the manufacturer’s protocol. After 48 h or 72 h transfection, cells were harvested and lysed to evaluate the transfection efficiency.

### Lentiviral vectors and viral infection

pHBLV-S100A13 and pHBLV-S100A13 mutant vectors were constructed by cloning the full-length S100A13 and S100A13 mutant fragments into the EcoRI/BamHI sites of pHBLV-puro vector. The two pLenti-shS100A13 vectors were constructed by cloning shRNAs against S100A13 which had the same sequences as two siRNAs into the HpaI/XhoI sites of pLentiLox 3.7-puro vector. Lentiviral constructs were transfected with packaging plasmids to HEK293T cells. The supernatant containing lentiviral particles was harvested at 48 h and filtered through a 0.45 μm filter, and then directly added to the culture medium. The infected cells were then selected with puromycin.

### Immunoblotting

Cell pellets were lysed in RIPA buffer (Applygen Technologies) containing phosphatase inhibitor (Roche Diagnostics) and protease inhibitor (Fermentas). Protein concentration was measured using the BCA Protein Assay Kit (Pierce). Cell lysates (20–40 μg) were subjected to 8–15% SDS-PAGE and transferred to nitrocellulose membranes (Millipore). The membrane was blocked using 5% milk in TBST buffer at room temperature for 1 h. Primary antibodies were blotted using 5% milk or BSA in TBST, and incubated at 4 °C overnight. The HRP-conjugated anti-mouse or anti-rabbit secondary antibodies were incubated for 1 h at room temperature in 5% milk/TBST. Then the signals were detected by enhanced chemiluminescence ECL (Pierce, Thermo Scientific), and imaged by films.

### FACS-based detection of membrane-bound IL-1α

For detection of membrane-bound IL-1α, 2.5 × 10^5^ cells were washed twice with PBS, then detached using 10 mM EDTA/PBS. Cells were collected, and washed twice with 0.5% BSA/PBS, then blocked with human IgG, and followed by incubation with FITC-labeled monoclonal antibodies against IL-1α (10 uL/1.0 × 10^5^ cells) for 1 h in the dark. After washing twice with 0.5% BSA/PBS and resuspending in 400 uL PBS, cells were processed by FACS analysis to determine the amount of cell surface-bound IL-1α using BD flow cytometer [11, 30].

### Real-time PCR

Total RNA was extracted using the RNeasy Mini kit (Qiagen) following the manufacturer’s protocol and then subjected to reverse transcription using the StarScript first strand cDNA synthesis kit (Transgen Biotech, Beijing, China). Real-time PCR was performed using SYBR Select Master Mix (Applied Biosystems) on an ABI PRISM 7500 Sequence Detector (Applied Biosystems). GAPDH served as an internal control for normalization [40].

The primers for RT-qPCR are listed as below:

S100A13 forward:

5’-TCCTAATGGCAGCAGAACCACTGA-3’ and

reverse:

5’- TTCTTCCTGATTTCCTTGGCCAGC-3’

IL-6 forward:

5’- TACCCCCAGGAGAAGATTCC -3’ and

reverse:

5’- TTTTCTGCCAGTGCCTCTTT -3’

IL-8 forward:

5’- TAGCAAAATTGAGGCCAAGG -3’ and

reverse:

5’- AAACCAAGGCACAGTGGAAC -3’

CXCL2 forward:

5’- GCCCAACGCACCGAATAGT-3’ and

reverse:

5’- CGCTGCCCATCATCATGAC-3’

CCL2 forward:

5’- AGTTCTTGCCGCCCTTCT -3’ and

reverse:

5’- GTGACTGGGGCATTGATTG -3’

IL-1β forward:

5’- GGGCCTCAAGGAAAAGAATC -3’ and

reverse:

5’- TTCTGCTTGAGAGGTGCTGA -3’

MMP3 forward:

5’- GCAGTTTGCTCAGCCTATCC -3’ and

reverse:

5’- GAGTGTCGGAGTCCAGCTTC -3’

IL-7 forward:

5’- CGCAAGTTGAGGCAATTTCT -3’ and

reverse: 5’- CTCTTTGTTGGTTGGGCTTC -3’

CXCL1 forward:

5’- AGGGAATTCACCCCAAGAAC -3’ and

reverse:

5’- TGGATTTGTCACTGTTCAGCA -3’

GAPDH forward:

5’- CGACCACTTTGTCAAGCTCA -3’ and

reverse:

5’- AGGGGTCTACATGGCAACTG -3’

### Inductively coupled plasma mass spectrometry (ICP-MS)

For detection of intracellular Cu^2+^ by ICP-MS, 1 × 10^7^ cells were washed twice with PBS, harvested and pelleted. Then the intracellular Cu^2+^ was measured by ICP-MS according to the manufacturer’s instructions.

### SA-β-Gal activity assay

SA-β-gal staining was performed according to the manufacturer’s protocol (Beyotime, Shanghai, China). Cells were washed twice with ice-cold 1 × PBS, and fixed in 3% formaldehyde for 10 min at room temperature. After washing twice with 1 × PBS, cells were stained at 37°C overnight in SA-β-gal staining solution without CO_2_, then images were acquired by photomicrography. 500 cells were counted to calculate the percentages of cells positive for SA-β-gal staining.

### Colony formation assay

To perform colony formation, 1 × 10^3^, 3 × 10^3^, and 1 × 10^4^ cells were cultured in six-well plate. After 10–14 days, cells were fixed in 3% (wt/vol) formaldehyde at 37 °C for 30 min and washed twice with 1 × PBS, then stained with crystal violet for 1h and washed twice with 1 × PBS followed by photography.

### Cell growth curves

Cell proliferation was detected using 2-(2-Methoxy-4-nitrophenyl)-3-(4-nitrophenyl)-5-(2,4-disulfophenyl)-2H-tetrazoliumsodiumsalt (CCK-8/WST-8) method. 2 × 10^3^ cells per well were seeded into 96-well plate and cultured for periods ranging from 1 to 6 day. The medium was changed every 24 h. At the indicated times, an aliquot of cells was stained with 10 μL of CCK-8 solution (Dojindo) for 2 h, and then the optical density at 450 nm was determined.

### Statistical analysis.

Two-tailed unpaired Student t test was used to determine the significance of differences between samples indicated in figures. Results are depicted as mean values ± standard deviation (SD, n=3). *P* < 0.05 (*), *P* < 0.01 (**), *P* < 0.005 (***), or *P* < 0.001 (****) were considered significant.

## SUPPLEMENTARY MATERIAL

Supplementary Figures
