# Supplementary material for: S100A13 promotes senescence-associated secretory phenotype and cellular senescence via modulation of non-classical secretion of IL-1α
Source: Aging (Albany NY). 2019 Jan 23;11(2):549–72. doi: 10.18632/aging.101760 (PMC6366962; doi:10.18632/aging.101760)
Supplement: Supplementary Figures [file aging-11-101760-s001.pdf]

SUPPLEMENTARY MATERIAL

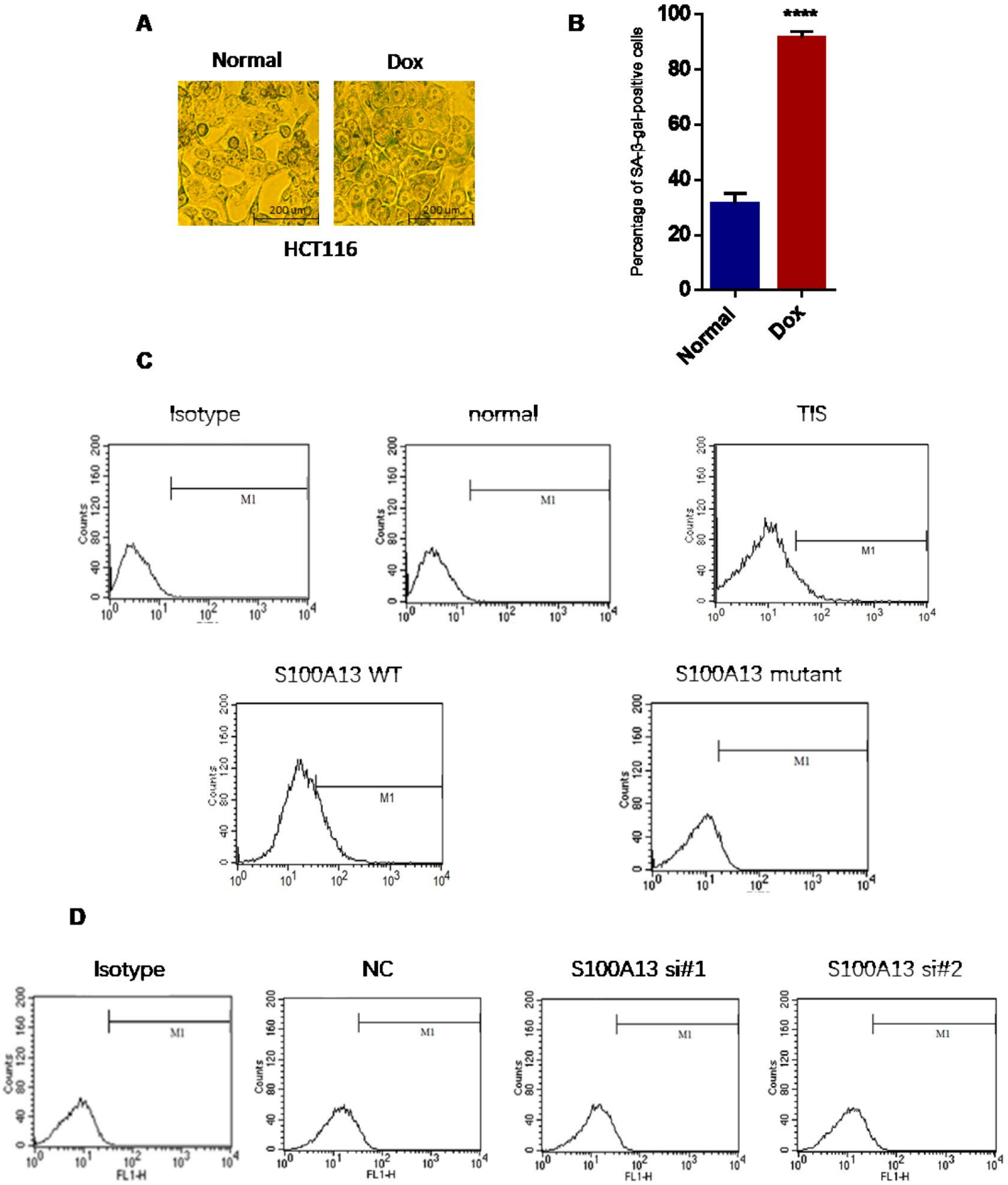

Supplementary Figure 1. Characterization of senescent state of TIS by SA-β-gal staining and measurement of cell surface-bound IL-1α levels by FACS (related to Figure 1). (**A** and **B**) Normal HCT116 cells either untreated or treated with 100 nM Dox for 4 days. Then cells were stained for SA-β-gal activity. The percentages of cells positive for SA-β-gal were calculated and graphed (n=3). (**C** and **D**) The indicated HCT116 cells were collected, washed, incubated in PBS with FITC-labeled monoclonal antibodies against IL-1α, and processed by FACS analysis to determine the amount of cell surface-bound IL-1α. Three independent experiments were analyzed. Error bars represent means ±SD (n = 3) *P < 0.05, **P < 0.01, ***P < 0.005, ****P < 0.001.

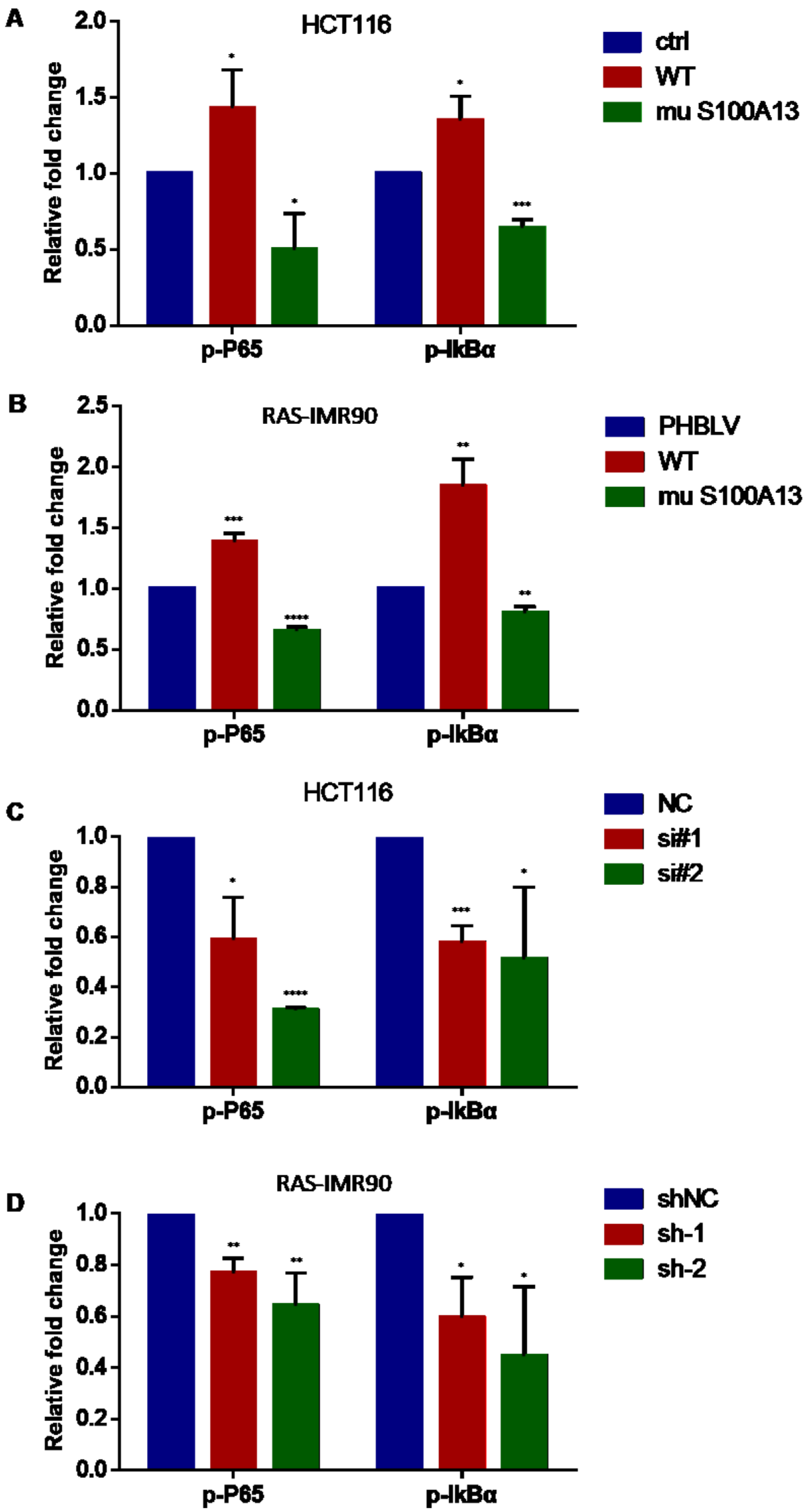

Supplementary Figure 2. Statistical analysis of p-p65 and p- IκBα levels (related to Figure 2A-D). S100A13 overexpression enhances p-p65 and p- IκBα levels, whereas S100A13 mutant or silencing decreases p-p65 and p- IκBα levels. Results are depicted as mean values ± SD (n=3). The data are presented as x-fold induction compared with untreated conditions (*). Statistical analysis was performed using Student's t-test. *P < 0.05, **P < 0.01, ***P < 0.005, ****P < 0.001 were considered significant.

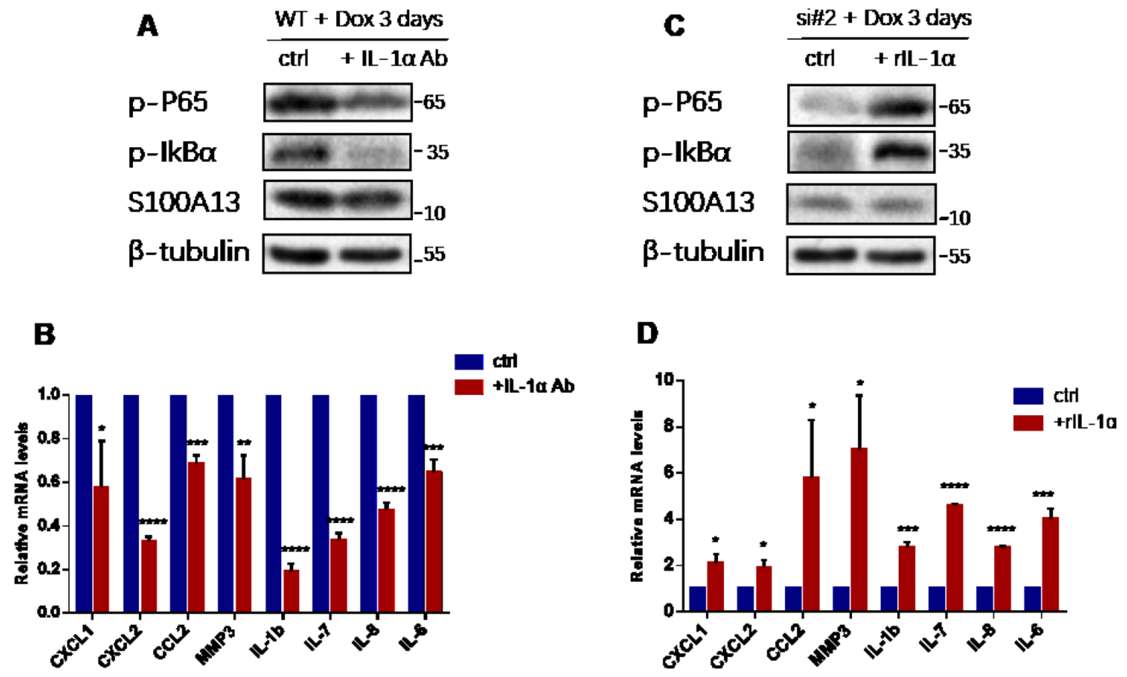

Supplementary Figure 3. Cell surface-bound IL-1α is required for S100A13 to regulate NF-κB activity and SASP induction during cell senescence (related to Figure 2G, H). (**A** and **B**) HCT116 cells were transfected with wild type S100A13, and treated with Dox (100 nM) for 3 days. Control IgG (0.6 ug/ml) or neutralizing antibody IL-1α (0.6 ug/ml) were added for the last 2 days. Then the indicated proteins were detected by western blot and mRNA levels of some SASP genes were analyzed by real-time qPCR (n=3). (**C** and **D**) HCT116 cells were transfected with siRNA#2 against S100A13, and treated with Dox (100 nM) for 3 days. Solvent or recombinant IL-1α protein (300 ng/ml) were added for the last 2 days. Then the indicated proteins were detected by western blot and mRNA levels of some SASP genes were analyzed by real-time qPCR (n=3). Three independent experiments were performed. Error bars represent means ± SD (n = 3) *P < 0.05, **P < 0.01, ***P < 0.005, ****P < 0.001 in (B) and (D).

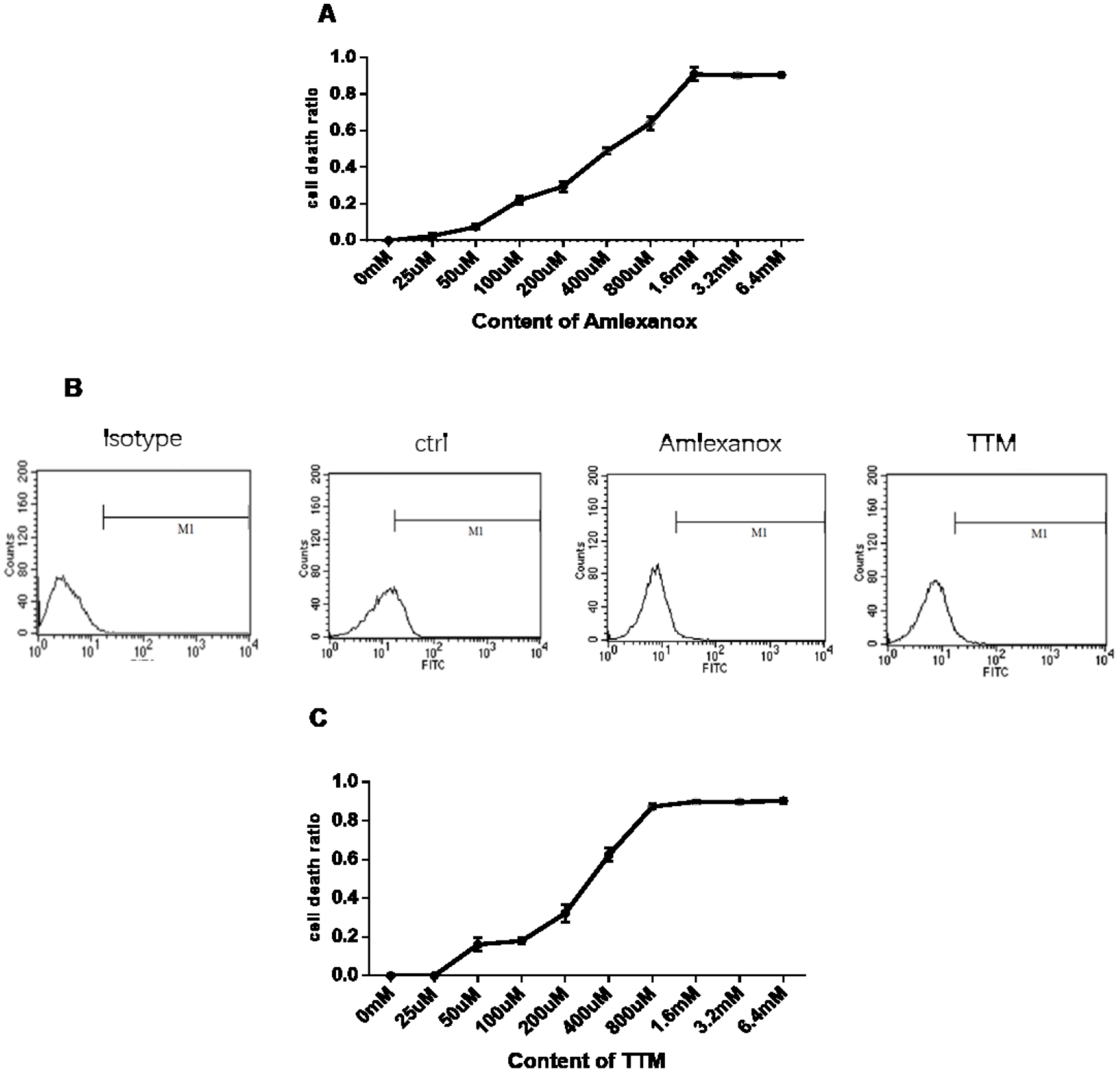

Supplementary Figure 4. Cell viability assay of Amlexanox and TTM to HCT116 cells and measurement of cell surface-bound IL-1α levels by FACS (related to Figure 3). (**A** and **C**) HCT116 cells were treated with the indicated doses of Amlexanox or TTM for 24 hours, then medium was removed and fresh medium was added to cells and CCK-8 assay was performed to measure the OD value of each sample. (**B**) HCT116 cells were treated with Dox (100 nM) for 4 days in the presence of Amlexanox or TTM. Then, cell surface-bound IL-1α were analyzed by FACS.

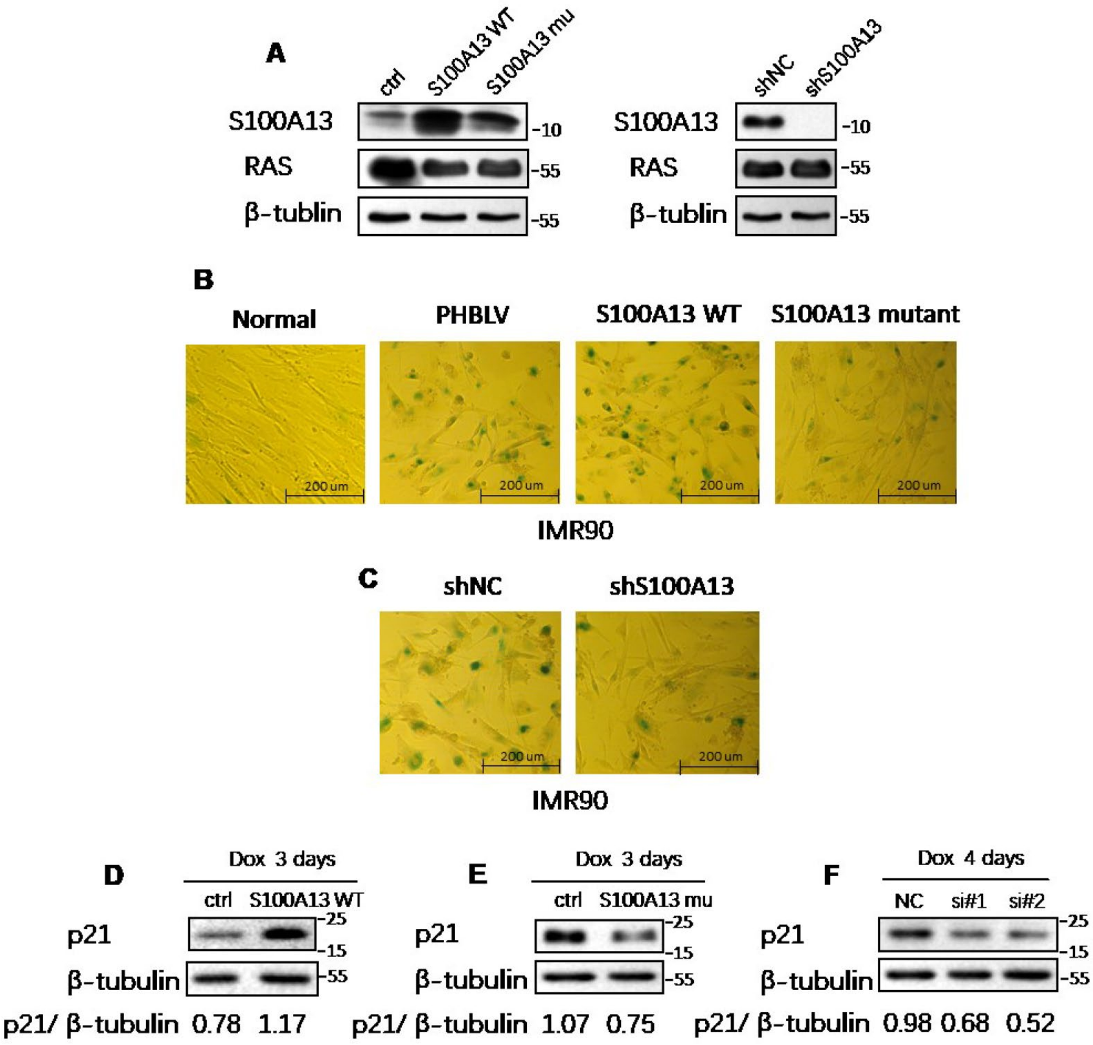

Supplementary Figure 5. S100A13 regulates Ras OIS and TIS (related to Figure 4). (**A**–**C**) Normal IMR90 cells were infected with lentivirus carrying oncogenic RAS to induce OIS, and at the same time cells were co-infected with a control vector (PHBLV), S100A13 wild type or mutant type (**B**), or control vector (shNC), shRNA against S100A13 (**C**). After 6 days, cell lysates were subjected to western blot analysis for the indicated proteins (**A**); and cells were stained for SA-β-gal activity (**B** and **C**). (**D**–**F**) HCT116 cells were transfected with the control, S100A13 wild type or mutant type (**D** and **E**), or transfected with the control (siNC) or two independent siRNAs against S100A13 (**F**), and treated with Dox (100 nM) for 3 or 4 days. Then the indicated proteins were detected by western blot.

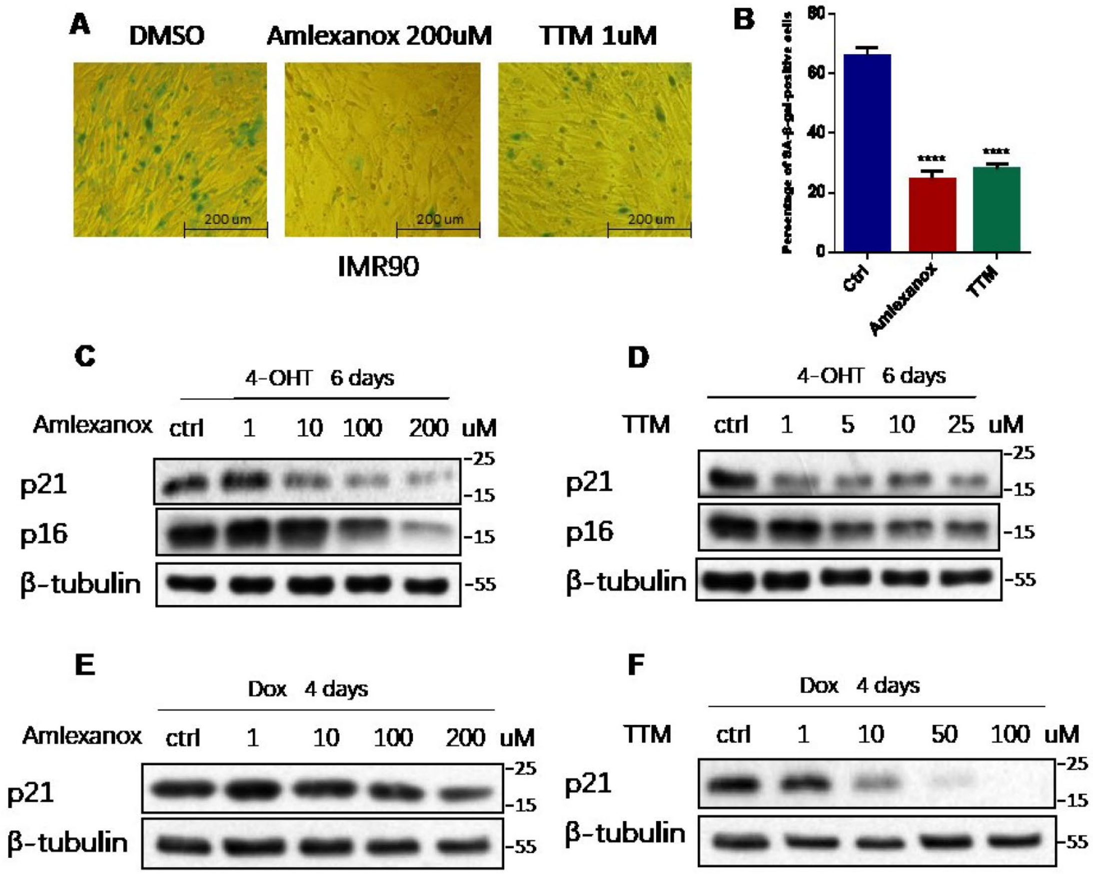

Supplementary Figure 6. Amlexanox and TTM modulates Ras OIS and TIS (related to Figure 4). (**A** and **B**) Normal IMR90 cells were infected with lentivirus carrying oncogenic RAS to induce OIS, and at the same time cells were treated with DMSO (control), TTM (1 uM) or Amlexanox (200 uM). After 6 days, cells were stained for SA-β-gal activity. The percentages of cells positive for SA-β-gal were calculated and graphed (n=3). ****P < 0.001. (**C** and **D**) ER:Ras IMR90 cells were given 4-OHT for total 6 days in the presence of the indicated doses of Amlexanox (**C**); or TTM (**D**); then the indicated proteins were analyzed by Western blot. (**E** and **F**) HCT116 cells were treated with Dox (100 nM) for 4 days in the presence of the indicated doses of Amlexanox or TTM. Then, the indicated proteins were detected by western blot.

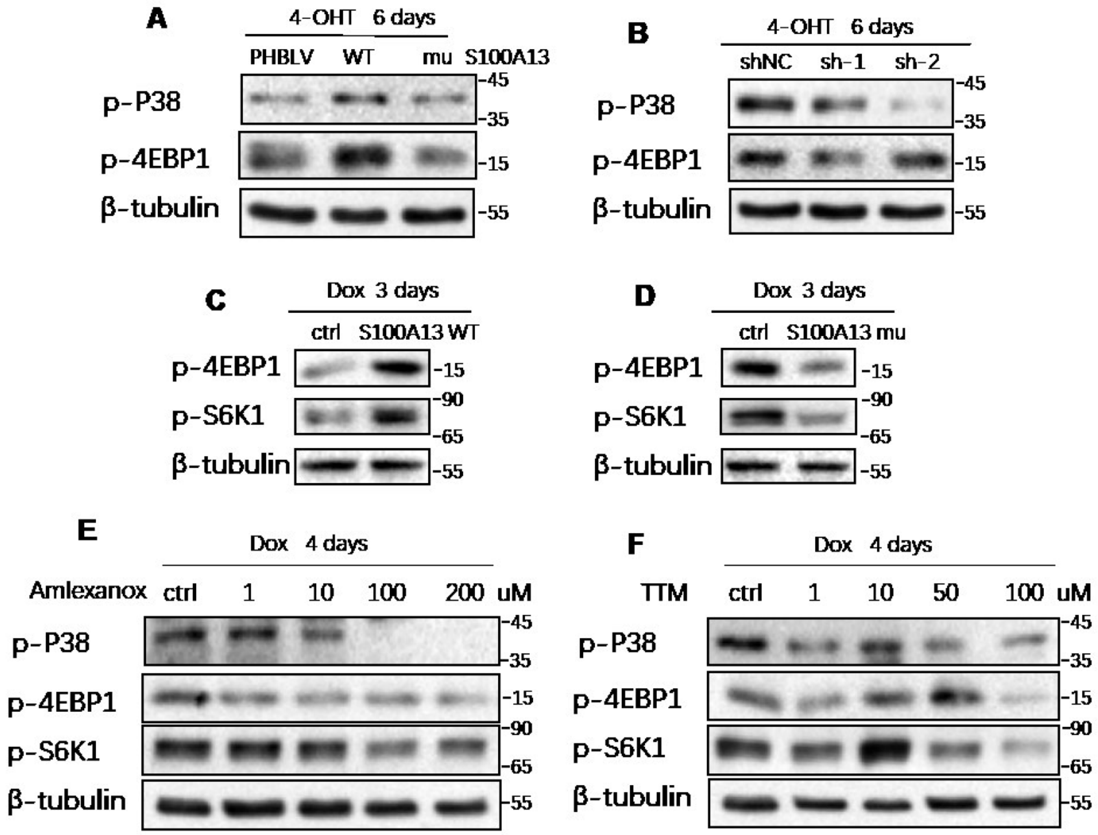

Supplementary Figure 7. Non-classical secretory pathway of IL-1α involves in regulating multiple SASP and cellular senescence regulators (related to Figure 6). (**A** and **B**) ER:Ras IMR90 cells stably transduced with PHBLV, S100A13 wild type or mutant type, or transduced with control (siNC) or two independent shRNAs against S100A13, were given 4-OHT for 6 days. Then the indicated proteins were detected by western blot. (**C** and **D**) HCT116 cells were transfected with the control, S100A13 wild type or mutant type, and treated with Dox (100 nM) for 3 days. Then the indicated proteins were detected by western blot. (**E** and **F**) HCT116 cells were treated with Dox (100 nM) for 4 days in the presence of the indicated doses of Amlexanox or TTM. Then, cell lysates were subjected to western blot analysis for the indicated proteins.
